# Mitochondrial localization of *Dictyostelium discoideum* dUTPase mediated by its N-terminus

**DOI:** 10.1186/s13104-019-4879-7

**Published:** 2020-01-07

**Authors:** Catherine P. Chia, Noriko Inoguchi, Kyle C. Varon, Bradley M. Bartholomai, Hideaki Moriyama

**Affiliations:** 1 School of Biological Sciences, Univ. Nebraska-Lincoln, Lincoln, NE 68588-0118 USA; 2 Department of Biological Sciences, Univ. Alabama in Huntsville, Huntsville, AL 35899 USA; 3 iXpressGenes Inc, Hudson Alpha Institute for Biotechnology, 601 Genome Way, Huntsville, AL 35806 USA; 4 Present Address: Geisel School of Medicine, Dept. Molecular and Systems Biology, Dartmouth College, Hanover, NH 03755 USA

**Keywords:** dUTPase, *Dictyostelium discoideum*, GFP, Mitochondrial targeting sequence

## Abstract

**Objective:**

The nuclear and mitochondrial genomes of *Dictyostelium discoideum*, a unicellular eukaryote, have relatively high A+T-contents of 77.5% and 72.65%, respectively. To begin to investigate how the pyrimidine biosynthetic pathway fulfills the demand for dTTP, we determined the catalytic properties and structure of the key enzyme deoxyuridine triphosphate nucleotidohydrolase (dUTPase) that hydrolyzes dUTP to dUMP, the precursor of dTTP.

**Results:**

The annotated genome of *D. discoideum* identifies a gene encoding a polypeptide containing the five conserved motifs of homotrimeric dUTPases. Recombinant proteins, comprised of either full-length or core polypeptides with all conserved motifs but lacking residues 1-37 of the N-terminus, were active dUTPases. Crystallographic analyses of the core enzyme indicated that the C-termini, normally flexible, were constrained by interactions with the shortened N-termini that arose from the loss of residues 1-37. This allowed greater access of dUTP to active sites, resulting in enhanced catalytic parameters. A tagged protein comprised of the N-terminal forty amino acids of dUTPase fused to green fluorescent protein (GFP) was expressed in *D. discoideum* cells. Supporting a prediction of mitochondrial targeting information within the N-terminus, localization and subcellular fractionation studies showed GFP to be in mitochondria. N-terminal sequencing of immunoprecipitated GFP revealed the loss of the dUTPase sequence upon import into the organelle.

## Introduction

The nuclear and mitochondrial genomes of *Dictyostelium discoideum* are 78% and 73% AT, respectively [1, 2], creating a substantial requirement for dUMP, the precursor for dTTP, during mitotic cell growth as well as during development when DNA replication also occurs [3–5]. To understand how the pyrimidine biosynthesis pathway accommodates the demand for dTTP, we began to focus on a key enzyme of the pathway, deoxyuridine triphosphate nucleotidohydrolase or dUTPase, which hydrolyzes dUTP to pyrophosphate and dUMP; dUMP is subsequently converted to dTTP. Concomitantly, a high dTTP to dUTP ratio is ensured, thus minimizing the incorporation of uracil during DNA synthesis [6].

**Fig. 1:**
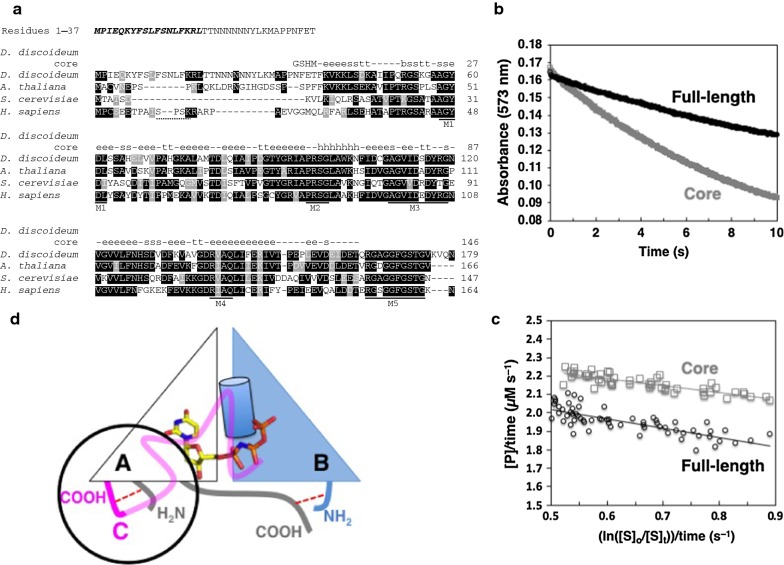
Recombinant full-length and core proteins were active dUTPases. **a** Alignment of polypeptide subunit sequences of homotrimeric dUTPases from eukaryotes and location of conserved motifs. Sequences used are: *Dictyostelium discoideum* (UniProt ID, Q54BW5), *Arabidopsis thaliana* (Q9STG6), *Saccharomyces cerevisiae* (P33317), *Homo sapiens* nuclear isoform 2, nuclear type (P33316-2). The human mitochondrial dUTPase isoform is not shown due to the lack of sequence similarity between its the N-terminal 69-residue targeting sequence and the *D. discoideum* N-terminus. The N-terminal Gly-Ser-His-Met (GSHM) of the *D. discoideum* core dUTPase is a result of the cloning process. Dashes (−) in sequences are alignment gaps by MAFFT [27] and the graphical output was generated by BoxShade [28]. In the human dUTPase, the sequence SPSK (dotted underline) is a consensus sequence for phosphorylation [29]. M1–M5 are five conserved motifs (solid underlines) in homotrimeric dUTPases [8]. The secondary structure composition of chain B in the *D. discoideum* core dUTPase is shown by lowercase letters in the top line. These were identified by the DSSP in the 3D-structure (PDB ID 5F9K) [30, 31] [29, 30]: ‘h’ = α-helix; ‘b’ = residue in isolated β-bridge; ‘e’ = extended strand; ‘t’ = turn; and ‘s’ = bend. Separately and above the alignment are shown residues 1–37 absent from the *D. discoideum* core dUTPase with a predicted MTS in bold italics [15–17]. **b** Estimation of kinetic parameters of recombinant full-length and core dUTPases. Example data sets (one of five independent measurements each) from stopped-flow spectroscopy used to monitor the decreasing absorbance of cresol red from protons released during hydrolysis of dUTP by either full-length (black) or core (gray) dUTPase, each at 0.15 µM. **c** Transformed absorbance data of Panel b yielded values for V_max_ and K_M_ of the full-length and core dUTPases (see Table 1) [32, 33]. d. Schematic illustration of the constrained orientations of the C-termini of Chains A and C of the core dUTPase. Triangles represent Chains A (white) and B (blue). A red dashed line shows the interaction between the C-terminus of Chain A (grey) and the N-terminus of Chain B (blue). Also shown with a red dashed line is the interaction between the C-terminus of Chain C (solid pink) and the N-terminus of Chain A (grey). This circled region is shown in more detail in Additional file 3: Fig. S3. Due to the lack of electron density, the C-terminus of Chain C (light pink) represents the region modeled on the high sequence identity to the *A. thaliana* dUTPase with its completed C-terminal coordinates (PDB ID 4OOP)

The curated genome of the soil amoeba *D. discoideum* shows a single gene (DictyBase Gene ID DDB_G0293374; [7]) predicted to encode a dUTPase polypeptide containing the five hallmark motifs (M1–M5) of homotrimeric dUTPases [8], seen in the alignments of the amino acid sequences from mustard, yeast and human (Fig. 1a). While the dUTPases of *Arabidopsis thaliana* and *D. discoideum* have substantial stretches of identity (73%) within the 138-residue segment containing M1–M5 [9], their N-termini have very low sequence similarity to each other, and to the human and yeast N-termini. Notably, within the lengthy N-terminus of the *D. discoideum* dUTPase, atypical of most dUTPases, computational analyses predict a mitochondrial targeting sequence (MTS).

## Main text

### Results

#### A single gene codes for an active enzyme in the homotrimeric dUTPase family

To characterize the unusual *D. discoideum* dUTPase, we established first that the recombinant protein was an active dUTPase and determined its kinetic parameters. Also catalytically-active was a core version comprised of polypeptide subunits that retain M1–M5 but lack residues 1-37 of the N-terminus. A homotrimeric structure of the core dUTPase enzyme was confirmed by crystallographic analyses that additionally showed interactions of the shorter N-termini with the C-termini likely enhanced substrate access of the dUTP substrate to the active sites.

Recombinant His-tagged dUTPase proteins were expressed in *E. coli*, purified by metal-chelating chromatography and after removal of the His-tag, assayed for activity by measuring the release of protons with the pH indicator cresol red (Additional file 1: Text S1). As determined by the kinetic parameters calculated from stopped-flow measurements (Fig. 1b, c and Table 1), the k_cat_/K_M_ of the core protein, lacking N-terminal residues 1-37 (Fig. 1a), was 60-fold greater than that of the full-length species, indicating the core was a more efficient enzyme.

**Table 1:** Calculated kinetic parameters of *D. discoideum* dUTPases

	K_M_, μM	V_max_, μM s^−1^	k_cat_^a^, s^−1^	k_cat_/K_M_, µM^−1^s^−1^
Core	0.5 ± 0.1	1.4 ± 0.06	9.3	18.6
Full-length	1.0 ± 0.2	0.5 ± 0.03	3.3	0.3

^a^k_cat_ = V_max_/[E_T_]; [E_T_] = 0.15 μM

Activity assays using an end-point method showed the full-length and core dUTPases both were metal-dependent enzymes inhibited by EDTA. Mg^2+^ was identified as the optimal divalent cation (Additional file 2: Table S1), as seen with homotrimeric dUTPases [6]. Both proteins specifically used dUTP (≤ 1% hydrolysis of other dNTPs; data not shown) and exhibited optimal activities at 60 °C and pH 8 (Additional file 3: Fig. S1). The catalytically efficient core dUTPase indicated that the 37 N-terminal residues were not essential for activity.

The crystal structure of the core dUTPase showed it to be a homotrimer, indicating that the absent 37 N-terminal residues were dispensable for oligomer formation (Additional file 3: Fig. S2; PDB ID 5F9K). In most homotrimeric dUTPases, the C-termini, containing M5 which aids in coordinating the ligand at the active site, are flexible. In the core structure, PISA interaction analyses revealed that the shorter N-termini interacted with the C-termini of adjacent subunits, constraining their engagement with their respective active sites. The interactions are schematically shown for Chains A and C, and Chains B and A (Fig. 1d). The limited movement of the C-termini contributed to the different inhibitor coordinates in each active site of the core dUTPase (Additional file 3: Fig. S2, insets). Notable is the position of Inhibitor B. Interactions (hydrogen bonds and salt bridges) by three glutamates of the C-terminus of Chain C with residues of the N-terminus of Chain A (E120::H3+F5, E123::K5 and E126::K9) produced the most displaced orientation compared to Inhibitors A and C (detailed in Additional file 3: Fig. S3). The C-termini, which act normally as lids for the substrate binding pockets [10–12], instead were restricted in their movement, which may have allowed increased access of dUTP to the three active sites compared to the full-length protein with its extended N-termini. We postulate this to be why the core enzyme was more catalytically efficient in vitro compared to the full-length dUTPase.

#### The N-terminus of *D. discoideum* dUTPase contains a mitochondrial targeting sequence

**Fig. 2:**
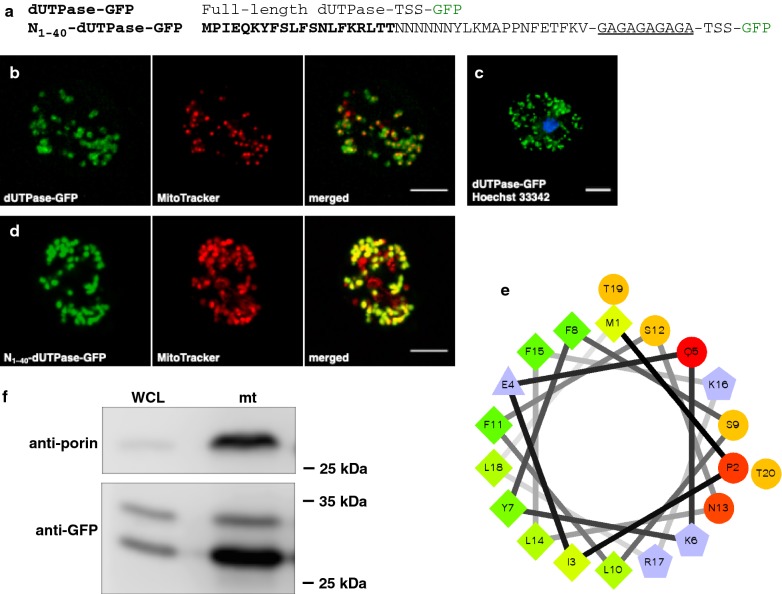
dUTPase-GFP fusion proteins localized to mitochondria. **a** Sequences of expressed *D. discoideum* dUTPase-green fluorescent protein (GFP) fusions. The full-length *D. discoideum* dUTPase polypeptide had GFP fused to the C-terminal Asn (N) 179 to produce dUTPase-GFP. Residues 1–40 of dUTPase were fused to GFP with a (Gly-Ala)_5_ linker; in bold are residues 1–20 used in the helical wheel drawn in Panel e. In both constructs, the Thr-Ser-Ser (TSS) tripeptide sequence arose from the cloning process. The underlined linker sequence was identified by N-terminal sequencing of protein immunoprecipitated by anti-GFP from Ax2 cells expressing N_1–40_-dUTPase-GFP. **b** Confocal microscopy images of live Ax2 cells expressing dUTPase-GFP stained with Mitotracker; merged signals showed co-localization. Scale bar, 5 µm. **c** Ax2 cells expressing dUTPase-GFP were fixed and stained with Hoechst 33342 to identify the nuclear DNA. dUTPase-GPF was absent from the nucleus. Scale bar, 5 µm. **d** Confocal microscopy images of live Ax2 cells expressing N_1–40_-dUTPase-GFP stained with Mitotracker; merged signals showed co-localization of the GFP signal with mitochondria. Scale bar, 5 µm. **e** Helical wheel of residues 1-20 of the N-terminus of *D. discoideum* dUTPase shows its amphipathic character. Symbols represent the following: circles, hydrophilic residues; diamonds, hydrophobic residues; triangles, potentially negatively charged; and pentagons, potentially positively charged. Color code: the most hydrophobic residue is green, and the amount of green is decreasing proportionally to the hydrophobicity, with zero hydrophobicity coded as yellow; hydrophilic residues are coded red with pure red being the most hydrophilic (uncharged) residue, and the amount of red decreasing proportionally to the hydrophilicity; potentially charged residues are light blue [13]. **f** Immunoblots showed N_1–40_-dUTPase-GFP in mitochondria. A mouse monoclonal specific for *D. discoideum* porin (upper panel; 30.1 kDa) [18] or a rabbit polyclonal anti-GFP (lower panel) was used to probe blots of whole cell lysates (WCL; ~ 100,000 cells) and enriched mitochondria preparations (mt; ~ 9 × 10^6^ cell equivalents) from Ax2 cells expressing N_1–40_-dUTPase-GFP. Positions of prestained markers are shown to the right of each blot

The role of the N-terminus of the *D. discoideum* dUTPase was explored first by expressing in Ax2 cells a full-length dUTPase with GFP at the C-terminus of the polypeptide subunit (Fig. 2a). By microscopy, the fusion protein was observed to localize to Mitotracker™–labeled mitochondria (Fig. 2b). This finding supported the hypothesis that dUTPase is a mitochondrial protein. It also raised the possibility that an MTS resides within the N-terminus. Contrary to expectations, nuclei did not display any GFP fluorescence (Fig. 2c). A helical wheel projection showed that N-terminal residues 1–20 of dUTPase formed an amphipathic helix [13] (Fig. 2e), with a net positive charge, a characteristic common to presequences of imported mitochondrial proteins [14]. Programs predicting targeting sequences indicated a high probability of dUTPase to be located in mitochondria, when fungi were used in the organism category, with cleavage after Leu18 upon import into the organelle (BaCelLo [15], MitoFates [16], MitoProt II [17]).

As a test for the presence of an MTS within the N-terminus of dUTPase, we added the forty N-terminal residues of dUTPase to GFP, producing N_1–40_-dUTPase-GFP (Fig. 2a). Like the full-length dUTPase-GFP fusion protein, the expressed N_1–40_-dUTPase-GFP co-localized with Mitotracker™–labeled mitochondria (Fig. 2d), reinforcing the prediction of an MTS within the N-terminus of dUTPase. The majority of the mitochondria had both GFP and Mitotracker™ signals, but not all Mitotracker™–stained mitochondria had a GFP signal. No fluorescence was observed in nuclei (data not shown).

Protein blots, of whole cell lysates and mitochondria prepared from cells expressing N_1–40_-dUTPase-GFP, were probed with antibodies against porin, a mitochondria-specific protein [18] or GFP (Fig. 2f). As expected, the signal for anti-porin was enhanced in the mitochondria preparation (upper panel). Observed also in mitochondria was a strong anti-GFP signal (lower panel) that corroborated the microscopy images, indicating that N_1–40_-dUTPase-GFP localized to mitochondria. The two anti-GFP signals observed both in the lysate and mitochondria were interpreted to be the entire N_1–40_-dUTPase-GFP, predicted to be 32.5 kDa and a processed version of it, migrating at 27.5 kDa. The approximate size difference of 5 kDa corresponded to the predicted 4.8 kDa mass of the forty residues of dUTPase fused to GFP, suggesting the loss of the dUTPase amino acids after import into the organelle.

To determine that cleavage of N_1–40_-dUTPase-GFP occurred, we obtained the N-terminal sequence of the 27.5 kDa protein immunoprecipitated from cell lysates using anti-GFP antibodies. The processed fusion protein was cleaved after Gly41, the first glycine of the (Gly-Ala)_5_ linker between the dUTPase and GFP sequences (Fig. 2a), confirming the loss of all forty dUTPase residues. Though within the range of presequence lengths, this was lengthier than the predicted presequence that identified cleavage after Leu18, a residue common within an R-2 motif recognized by the mitochondrial processing peptidase [16, 19] (reviewed in 14). An intermediate was not detected by immunoblotting, but it is possible that the fusion protein was cleaved twice, once after Leu18 and then after Gly41, generating the final A(GA)_4_-GFP molecule inside the mitochondria.

#### Summary

Activity and structural analyses of the *D. discoideum* dUTPase showed it to be a functional enzyme with attributes typical of most eukaryotic dUTPases. The unusually long N-terminus of the polypeptide subunit was found to be dispensable for catalytic activity as well as homotrimer formation. Microscopy and biochemical evidence showed that fusion proteins, where GFP was attached to the full-length polypeptide or the forty N-terminal amino acids of dUTPase, localized to mitochondria, but not to nuclei, indicating the presence of an MTS within the N-terminus of the *D. discoideum* dUTPase.

## Limitations

The presence of dUTPase in *D. discoideum* mitochondria is reasonable given the needed synthesis of nucleotides for replication of the organellar DNA that is 73% AT. Except for the human enzyme, however, no other dUTPase has been reported to be in mitochondria. Based on its nuclear presence in yeast, flies and humans [20–23], we also expected to see dUTPase in nuclei, but did not observe a GFP signal in nuclei of *D. discoideum* cells of log-phase cultures expressing the full-length dUTPase-GFP fusion protein (Fig. 2c). Technical reasons could account for an absence of a nuclear signal. Fused to the C-terminus and larger than an individual dUTPase subunit (mass of the predicted full-length polypeptide is 19.8 kDa), the GFP sequence (27.6 kDa) may have interfered with homotrimer assembly or masked nuclear localization signals. The successful cytological detection of dUTPase in the nucleus may require a construct with a small tag that is less likely to disrupt folding or cover signal epitopes on the dUTPase surface, coupled with methods that synchronize cell division or identify cells entering S-phase when presumably the nucleotide biosynthesis enzymes are actively providing dNTPs for DNA replication.

Alternatively, a nuclear-localized dUTPase in *D. discoideum* may be a location-specific isoform, rather than the predicted full-length protein. The human dUTPase has nuclear and mitochondrial isoforms produced from the same gene through the use of alternative 5′ exons [23, 24]. In *Drosophila*, two transcripts for dUTPase also are produced from a single gene, resulting in two isoforms with different N-termini [20]. Depending on the tissue and developmental stage, the isoforms are present either in the nucleus or the cytoplasm; neither is found in mitochondria. Currently, there are no data (such as cDNAs) pointing to *D. discoideum* dUTPase isoforms, but these cannot be ruled out as there are examples in *D. discoideum* where two versions of a protein are generated from single copy genes. These include the peroxisomal and mitochondrial isoforms of the enzyme acetoacetyl-CoA thiolase (DDB_G0271544) that arise from alternative start codons [25], and (cytoplasmic and mitochondrial) isoforms of fumarase (DDB_G0280495), inferred from expressed sequence tag data. Fumarase isoforms have been documented extensively in yeast and mammals (reviewed in 26).

An increasing number of eukaryotic proteins are reported to have more than one subcellular location, and enzymes of nucleotide synthesis pathways are candidates for multiple locations since DNA replication occurs in the nucleus, mitochondria and chloroplasts. The mitochondrial location of the *D. discoideum* dUTPase, and the previously identified mitochondrial and nuclear isoforms of the human dUTPase, raises questions of whether other enzymes in the pyrimidine nucleotide biosynthesis pathway are similarly positioned and whether other eukaryotes share this pattern of multiple locations. The genetically-tractable *D. discoideum* is an experimental system that can be exploited to determine and explore molecular mechanisms of the subcellular distribution of thymidylate synthase, dCMP and dCTP deaminases (supported by expressed sequence tag data), and should reveal whether these enzymes use targeting strategies that coordinate their distribution with that of dUTPase, ensuring dNTPs for the replication of nuclear and organellar DNA.

## Supplementary information

**Additional file 1: Text S1.** Methods.

**Additional file 2.** Metal-dependence and statistics for refinements.

**Additional file 3: Figure S1.** Temperature and pH optima. **Figure S2.** Secondary structure & altered positions. **Figure S3.** Interactions between Chains A and C.

## Data Availability

The structure of the *D. discoideum* core dUTPase was deposited in the Protein Data Bank with the ID 5F9K. The dataset supporting the conclusions of this article are included within the article and additional materials.

## Abbreviations

dUTPase: deoxyuridine triphosphate nucleotidohydrolase
GFP: green fluorescent protein
MTS: mitochondrial targeting sequence
