# Supplementary material for: Mitochondrial localization of Dictyostelium discoideum dUTPase mediated by its N-terminus
Source: BMC Res Notes. 2020 Jan 7;13:16. doi: 10.1186/s13104-019-4879-7 (PMC6947831; doi:10.1186/s13104-019-4879-7)
Supplement: Supplementary file 1 — Additional file 1: Text S1. Methods. [file 13104_2019_4879_MOESM1_ESM.docx]

**Additional Material Text S1 Methods**

**Molecular Cloning**

Standard cloning methods were used to generate the constructs for the His-tagged full-length and core dUTPases, a full-length dUTPase with green fluorescent protein (GFP) fused at the C-terminus (dUTPase-GFP), and the fusion of the forty N-terminal amino acids of dUTPase to the N-terminus of GFP (N_1-40_-dUTPase-GFP). Plasmid DNA was isolated using Miniprep and Midiprep kits (Qiagen, Inc., Valencia, CA), and digested DNA was gel-purified using a gel extraction kit (Qiagen). Restriction enzymes, antibiotics and buffers were purchased from ThermoFisher Scientific (Waltham, MA). Plasmids pDM313 and pDM304 were acquired from the Dicty Stock Center [1, 2].

**His-tagged *D. discoideum* dUTPases**

The DNA coding sequence corresponding to the predicted full-length dUTP nucleotidohydrolase (EC 3.6.1.23) polypeptide of *D. discoideum* (DictyBase Gene ID DDB_G0293374) was synthesized flanked with EcoRI and BamHI sites, in pUC57 (GenScript USA Inc., Piscataway, NJ). With this template, a PCR product generated with the sense (CCAGTGAACATATGCCAATCGAACAAAAATATTTTTC) and antisense (GGCCCGGGATCCTTAATTTTGAACTTTAACACC) primers, containing NdeI and BamHI restriction sites (underlined) respectively, was ligated into the corresponding sites of pET-15b (Novagen, San Diego, CA). The DNA sequence corresponding to amino acid residues 38-179 of *D. discoideum* dUTPase (‘core’ dUTPase) was synthesized flanked by BamHI and EcoRI sites, in pUC57 (GenScript). With this template, a PCR product generated with sense (CATATGTTCAAAGTTAAAAAATTAT) and antisense (GGATCCTTAATTTTGAACTTTAACA) primers, containing NdeI and BamHI restriction sites (underlined) respectively, was cloned into pGEMT (Promega Corp., Madison, WI). The sequence for the core dUTPase was released with NdeI and BamHI, and ligated into pET-15b.

For both His-tagged full-length and core dUTPase constructs, plasmid DNA from transformed DH5alpha cells (New England BioLabs, Ipswich, MA) was sequenced to confirm the insert (Sequetech Corp., Mountain View, CA) before using to transform competent BL21(De3)pLys bacteria (Invitrogen/ThermoFisher Scientific). Cultures of bacteria were grown at 35–37 ˚C in Luria-Bertani media containing 100 µg/mL ampicillin to an optical density of 0.6 at 600 nm. Expression of the His-tagged dUTPases was induced with 1 mM isopropyl β-D-1-thiogalactopyranoside. Cultures were grown for an additional four hours at 35 ˚C (full-length) or 18 h at 22 ˚C (core) before harvesting. Bacterial pellets were stored at -20 ˚C.

**Recombinant protein purification**

Bacteria (from a 250 mL culture) were suspended in 20 to 25 mL NP1-10 (50 mM NaH_2_PO_4_, 300 mM NaCl, 10 mM imidazole, pH 8.0) and sonicated at 4 ˚C with a Sonicator 3000 (Misonix Inc., Farmingdale, NY) for 10 minutes (20 s rest/10 s burst) at a power setting of 3.5. After centrifugation (12,000 x g, 60 min, 4 ˚C), the clarified lysate was loaded onto a HiTrap™ Chelating HP column (1 mL bed volume; GE Healthcare Life Sciences, Pittsburgh, PA) charged with NiCl_2_ (HiTrap Chelating HP 1 mL User Manual) and equilibrated with NP1-10. Purification was carried out with a BioCad Vision Workstation (Applied Biosystems, Framingham, MA) with a linear gradient of 0% to 100% NP1-250 (50 mM NaH_2_PO_4_, 300 mM NaCl, 250 mM imidazole, pH 8.0) at 1 mL/min. The loading rate was adjusted to 0.5 mL/minute to allow for binding of the His_6_-tagged protein. Clarified lysate was passed through the column five consecutive times to allow for total binding. The column was washed with NP1-20 (50 mM NaH_2_PO_4_, 300 mM NaCl, 20 mM imidazole, pH 8.0) for 20 minutes following binding. Protein was monitored by OD280 and typically a peak was observed 15 to 20 mL into the elution step (corresponding to 90 to 100%, NP1-250). Peak OD280 fractions were analyzed by SDS-PAGE and purity of Coomassie-stained protein was assessed using ImageJ (Image Processing and Analysis in Java, http://rsbweb.nih.gov/ij/). Peak protein fractions were filter-dialyzed (Amicon Ultra-4, 30,000 NMWL, EMD Millipore Billerica, MA) into phosphate buffered saline (PBS; 8.1 mM Na_2_HPO_4_, 1.9 mM NaH_2_PO_4_•H_2_O, 150 mM NaCl, pH 7.2). His_6_-tagged proteins were treated with bovine alpha-thrombin (10 Units/mg recombinant protein; Haematologic Technologies Inc., Essex Junction, VT) at 22°C for 2 to 4 h to remove the His_6_-tag. As a consequence of cloning, both the recombinant full-length and core dUTPase proteins have the tripeptide glycine-serine-histidine at the N-terminus. Benzamidine and phenylmethanesulfonylfluoride (Sigma-Aldrich, St. Louis, MO) were each added to a final concentration of 1 mM to inhibit thrombin activity. Alternatively, preparations were adjusted to 50 mM Tris-HCl, 500 mM NaCl, pH 7.4 and passed through 1 ml HiTrap™ Benzamidine FF columns. Loss of the His-tag was verified by SDS-PAGE. Filter-dialyzed, protein was concentrated into 10X Bicine buffer (2.5 mM bicine, 1 M KCl, 50 mM MgCl_2_, pH 8.0) and stored at 4˚C. Protein was quantified with a Pierce™ BCA Protein Assay Kit (ThermoFisher Scientific) using bovine serum albumin as a standard. An OD280 of 1.0 corresponded closely to 1 mg/mL, using the BCA assay, of the recombinant proteins without their His-tags.

**GFP-tagged dUTPase**

For the expression of a full-length dUTPase-GFP fusion protein, the coding sequence for full-length dUTPase polypeptide was synthesized in pUC57 (GenScript) flanked by SacI and SpeI sites at the 5’ and 3’ ends, respectively. The gene was excised with these enzymes and ligated into the corresponding sites in pDM313 to fuse it in frame with the sequence coding for GFP. The dUTPase-GFP construct was excised with BamHI and XbaI and ligated into expression plasmid pDM304 digested with BglII and SpeI.

The DNA coding sequence for residues 1-40 of the *D. discoideum* dUTPase was synthesized in pUC57 (GenScript) flanked at the 5’ end by restriction sites SacI followed by BamHI, and at the 3’ end, the sequence for a (Gly-Ala)_5_ spacer followed by SpeI-EcoRV sites. The sequence was excised from pUC57 with SacI and SpeI and ligated into the corresponding sites of pDM313, resulting in the fusion of the sequence of the 40 N-terminal amino acids of dUTPase and the linking (Gly-Ala)_5_ peptide to the sequence encoding GFP. The construct was released with BglII and SpeI, ligated into expression vector pDM304. Before transforming *D. discoideum* cells, plasmids were verified by sequencing (Sequetech).

**Kinetic Measurements**

Assays of dUTPase activity were measured as described [3, 4] using a stopped-flow spectrometer (Hi-Tech SF-61DX2, TgK Scientific, Bradford-on-Avon, UK) equipped with a photoarray detector. The degassed assay buffer solution contained 100 mM KCl, 5 mM MgCl_2_, 0.25 mM bicine and 25 µM cresol red, pH 7.6. Full-length or core dUTPase (0.15 µM final concentration), was rapidly mixed with assay buffer containing dUTP (7.5 µM final concentration) in the stopped-flow system (at 25˚C), and absorbance changes were monitored at 573 nm. The enzyme kinetic parameters K_M_ and V_max_ were determined using the integrated Michaelis-Menten equation [4].

**HPLC end point analyses of dUMP produced from hydrolysis of dUTP by recombinant dUTPases**

Anion exchange chromatography was performed with a BioCad Vision Workstation using three 1 mL HiTrap™ Q HP columns (GE Healthcare) in sequence to separate the substrate dUTP (Sigma-Aldrich) from the product dUMP. Recombinant full-length (0.25 µg; 1.4 nM) or core (0.5 µg; 3.6 nM) dUTPase was mixed into three mL of HPLC assay buffer (10 mM bicine, 2 mM MgCl_2_, pH 8.0) containing 10 µM dUTP pre-equilibrated in a water bath for 10 min at 25˚C (or different temperatures for analyses of optima). After 10 min., the reaction was quenched by the addition of 60 µL of 1N HCl. Two mL of the three mL reaction were analyzed per sample. Columns were equilibrated with 5 mM potassium phosphate, 10 mM KCl, pH 8.0 (Solution A). Reaction samples were loaded followed by 6 mL of 80% Solution A, 20% Solution B (5 mM potassium phosphate, 500 mM KCl, pH 8.0). Nucleotides were eluted with a linear gradient (2 mL/min) of 20% to 100% Solution B over 15 min. The absorbance at 260 nm was monitored and the amount of dUMP produced in two mL of the sample was obtained by measuring the area under the dUMP peak using the graphic display of chromatograms method editor (Vision Workstation Software V 3.01). Known amounts of dUMP (Sigma-Aldrich) in 10 mM bicine, pH 8.0 were chromatographed to generate a standard curve. Metal-dependence measurements (at 25˚C) used 10 µM dUTP, 10 mM bicine, pH 8.0 containing 2 mM MgCl_2_, MnCl_2_, CaCl_2_ or EDTA (SM Table S1). Substrate specificity trials used 10 µM dNTP (GE Healthcare) in the HPLC assay buffer. For pH optima measurements (25˚C), the assay buffers, containing 10 µM dUTP and 10 mM MgCl_2_, were: 10 mM citrate (pH 4.0, 5.0, 6.0), 10 mM HEPES (pH 7.0, 8.0), or 10 mM borate (pH 9.0, 10.0).

**Cell culture and protein analyses**

Ax2 *D. discoideum* cells were grown in HL5 media in shaken suspension cultures at 22˚C [5]. Cells were transformed by electroporation [6] with 10 µg of the pDM304 plasmid construct coding for either dUTPase-GFP or N_1-40_-dUTPase-GFP. Transformants were selected in HL5 media containing 20 µg/mL G418 and subcloned by dilution to obtain independent isolates. Enriched mitochondria preparations from Ax2 and transformants expressing N_1-40_-dUTPase-GFP were isolated from mid-log axenic cultures (4–6 x 10^6^ cells/mL) following the protocol described [7] except BSA was omitted and cells were lysed by passage through 3 µm Nuclepore® filters (GE Healthcare). SDS was added immediately to aliquots of lysed cells for protein gels.

Protein samples were analyzed by SDS-PAGE using the Laemmli buffer system [8]. Gels were stained using Coomassie blue (0.25 g/L Coomassie R-250, 50% methanol, 10% acetic acid). Protein standards (PageRuler Prestained Protein Ladder; ThermoFisher) were used to determine the relative mobility of recombinant proteins by plotting the log of the molecular mass of the standards against their migration distance in acrylamide gels or after transfer to blots. The core dUTPase polypeptide, predicted to be 15.5 kDa migrated as a 16 kDa species. The full-length dUTPase polypeptide, predicted to be 19.8 kDa, migrated as a 21.8 kDa species.

**Immunoblots and Immunoprecipitation**

For immunoblots, proteins separated on SDS-gels were transferred to polyvinylidene fluoride (PVDF) membranes (0.2 µm pore size; Bio-Rad) by semi-dry blotting (1.5 mA/cm^2^ for 1 h) using a discontinuous buffer system where the anode buffer was 40 mM N-cyclohexyl-3-aminopropanesulfonic acid (CAPS), 60 mM Tris pH 9.6, 15% methanol and the cathode buffer was 40 mM CAPS, 60 mM Tris, pH 9.6, containing 0.1% SDS (Bio-Rad Bulletin 2134). Blots were blocked with 5% non-fat milk in 25 mM Tris, 150 mM NaCl, pH 7.2 (TBS), incubated with primary antibodies diluted in 5% bovine serum albumin and washed with TBS containing 0.1% Tween-20 (TBST) and then incubated with either goat anti-rabbit or donkey anti-mouse horse radish peroxidase conjugates (Invitrogen/ThermoFisher Scientific) in 5% milk in TBS (1:5,000). After washing blots with TBST, immunodetection was performed using Clarity™ Western ECL substrate (Bio-Rad) with a C-DiGit Blot Scanner (Image Studio Software ver. 5.2.5; LI-COR Biosciences, Lincoln, NE). Monoclonal antibodies to GFP (DSHB-GFP-12A6 and DSHB-GFP-4C9) [9] and a monoclonal antibody recognizing porin (30 kDa) of *D. discoideum* [10] were acquired from the Developmental Studies Hybridoma Bank (created by the NICHD of the NIH and maintained at The University of Iowa, Department of Biology, Iowa City, IA). A rabbit polyclonal antiserum to GFP was from Invitrogen/ThermoFisher Scientific.

For immunoprecipitations, lysates of log-phase Ax2 cells expressing N_1-40_-dUTPase-GFP were prepared using 50 mM Tris, 150 mM NaCl, 1%NP-40, 0.5% sodium deoxycholate, 1 mM EDTA, 1 mM phenylmethylsulfonyl fluoride, pH 7.4. One ml of lysate (4–6 x 10^7^ cells/mL) was incubated with 50 µL of Dynabeads® Protein A or Protein G (Invitrogen/ThermoFisher Scientific) with bound anti-GFP antibodies following vendor protocols. After washing, the Dynabeads® were treated with gel sample buffer (62.5 mM Tris, pH 6.8, 1% SDS, 1 mM EDTA, 10% sucrose, 0.1% bromphenol blue; 10 min, 80 ˚C) to elute bound protein that then was run on SDS-gels (12.5% polyacrylamide). PVDF blots of gels were stained 5 min. with Coomassie blue (0.2% R-250, 20% methanol, 0.5% acetic acid), destained with 30% methanol and washed with water. N-terminal sequencing of blotted, immunoprecipitated protein from each of three independently prepared samples was performed by the Protein Structure Core Facility (U. Nebraska Medical Center, Omaha, NE).

**Microscopy**

*Live cell imaging*

Ax2 and Ax2 cells expressing dUTPase-GFP or N_1-40_-dUTPase-GFP were grown in FM media (Formedium, Norfolk, UK) supplemented with yeast extract (0.5 g/liter) in glass bottom dishes (Matsunami Glass, Bellingham, WA). Prior to imaging, the media was exchanged with FM media containing MitoTrackert™ Red FM (10 nM; LifeTechnologies/ThermoFisher) and incubated 1 h at 100 rpm on a rotary shaker. Two washes were performed with FM medium on the shaker after MitoTracker™ staining. Images were acquired with an Andor W1 Spinning Disk system on a Nikon Eclipse Ti-E microscope with Perfect Focus driven by Nikon Elements. Dual Andor Zyla 4.2 PLUS sCMOS cameras were used with a Chroma quad (405/445/514/785) excitation filter and Chroma 525/50 nm and 600/50 nm emission filters for simultaneous acquisition of green (488 nm) and red (561 nm) channels.

*Fixed cell imaging*

Ax2 cells expressing full-length dUTPase-GFP were grown to sub-confluence in glass bottom dishes. Cells were fixed for 10 minutes with methanol-free 4% formaldehyde in PBS and washed three times with PBS. Nuclear staining was carried out with Hoechst 33342 added to the second wash. Imaging was carried out on a Nikon Eclipse Ti-E widefield microscope driven by Nikon Elements, equipped with an Andor Zyla 4.2 PLUS sCMOS camera, a Lumencor Sola SE2 light engine, and Chroma filter sets 49028 (ET – DAPI for 395 nm light sources) and 49011(ET - FITC/Alexa Fluor 488/Fluo3/Oregon Green). Nikon Elements was used for image processing, including 15 iterations of deconvolution using the Richardson-Lucy algorithm in both channels. All images were converted to Tiff format and contrasted for display using FIJI.

**Crystallization, X-ray diffraction data collection and structure solution analyses**

The crystallization of the core dUTPase was achieved by the hanging-drop vapor-diffusion method [11] as previously described [3, 12] with slight modifications using an EasyXtal 15-Well Tool (Qiagen), the NeXtal crystallization apparatus. A 4 μl protein droplet containing 0.2 mM of the (core) dUTPase, 0.6 M ammonium sulfate (AS), 0.6 mM MgCl_2_, 0.6 mM 2’-deoxyuridine-5’-[(α,β)-imido]triphosphate (dUMPNPP; Jena Bioscience, Jena, Germany), and 25 mM potassium phosphate buffer, pH 7.0 was equilibrated in a 500 μl reservoir consisting of 1.5 M AS in the same buffer at 22˚C. After 8 days, the droplet was still clear, and the AS concentration was increased to 1.7 M by adding 84 μl of 3.0 M AS. The crystallization vial was kept at the same temperature. After ~120 days, a crystal was found on the inside of the skin.

Diffraction data were collected from a single holo (core) dUTPase crystal briefly soaked in a cryo-protectant consisting of 10 μl glycerol and 100 μl of the crystallization reservoir and mounted by a loop using a flush-freezing technique [13] under the cold stream at 100 K. Diffraction images were recorded by the oscillation method with a swing width of 1˚ using the ADSC Q315 detector (sector 14-BM-C) at the Advanced Photon Source BioCARS [14]. Diffraction images up to a resolution of 2.2 Å were indexed, integrated, and scaled using HKL2000 [15]. The initial phase was obtained by the molecular replacement method using PHENIX [16] with *Arabidopsis* dUTPase as the search model (Protein Data Bank ID 4OOP) [3]. The solution was subjected to iterative cycles of restrained refinements in PHENIX and manual model building in Coot [17]. The crystal belonged to the orthorhombic space group P2_1_2_1_2_1_ and the asymmetric unit contains a homotrimer with a solvent content of 50.6%. The structure was refined to R_work_ and R_free_ values of 21.0% and 25.0%, respectively (SM Table S2). The structure was deposited in the Protein Data Bank with the ID 5F9K.

Structural mining and graphics preparation were performed with The PyMOL Molecular Graphics System (Schrödinger, LLC). Interfaces of proteins were analyzed on the PISA web service [18].

**References**

1. Fey P, Dodson RJ, Basu S, Chisholm RL. One stop shop for everything *Dictyostelium*: dictyBase and the Dicty Stock Center in 2012. Methods Mol Biol. 2013;983:59-92.

2. Veltman DM, Akar G, Bosgraaf L, Van Haastert PJ. A new set of small, extrachromosomal expression vectors for *Dictyostelium discoideum*. Plasmid. 2009;61(2):110-8.

3. Inoguchi N, Chaiseeda K, Yamanishi M, Kim MK, Jang Y, Bajaj M, et al. Structural insights into the mechanism defining substrate affinity in *Arabidopsis thaliana* dUTPase: the role of tryptophan 93 in ligand orientation. BMC Res Notes. 2015;8(1):784.

4. Larsson G, Nyman PO, Kvassman JO. Kinetic characterization of dUTPase from *Escherichia coli*. J Biol Chem. 1996;271(39):24010-6.

5. Fey P, Kowal AS, Gaudet P, Pilcher KE, Chisholm RL. Protocols for growth and development of *Dictyostelium discoideum*. Nature protocols. 2007;2(6):1307-16.

6. Gaudet P, Pilcher KE, Fey P, Chisholm RL. Transformation of *Dictyostelium discoideum* with plasmid DNA. Nature protocols. 2007;2(6):1317-24.

7. Czarna M, Mathy G, Mac'Cord A, Dobson R, Jarmuszkiewicz W, Sluse-Goffart CM, et al. Dynamics of the *Dictyostelium discoideum* mitochondrial proteome during vegetative growth, starvation and early stages of development. Proteomics. 2010;10(1):6-22.

8. Laemmli UK. Cleavage of structural proteins during the assembly of the head of bacteriophage T4. Nature. 1970;227:680-5.

9. Sanchez P, Daniels KJ, Park YN, Soll DR. Generating a battery of monoclonal antibodies against native green fluorescent protein for immunostaining, FACS, IP, and ChIP using a unique adjuvant. Monoclon Antib Immunodiagn Immunother. 2014;33(2):80-8.

10. Troll H, Malchow D, Muller-Taubenberger A, Humbel B, Lottspeich F, Ecke M, et al. Purification, functional characterization, and cDNA sequencing of mitochondrial porin from *Dictyostelium discoideum*. J Biol Chem. 1992;267(29):21072-9.

11. McPherson A, Weickmann J. X-ray analysis of new crystal forms of the sweet protein thaumatin. J Biomol Struct Dyn. 1990;7(5):1053-60.

12. Bajaj M, Moriyama H. Purification, crystallization and preliminary crystallographic analysis of deoxyuridine triphosphate nucleotidohydrolase from *Arabidopsis thaliana*. Acta Crystallogr Sect F Struct Biol Cryst Commun. 2007;63(Pt 5):409-11.

13. Pflugrath JW. Macromolecular cryocrystallography--methods for cooling and mounting protein crystals at cryogenic temperatures. Methods. 2004;34(3):415-23.

14. Graber T, Anderson S, Brewer H, Chen YS, Cho HS, Dashdorj N, et al. BioCARS: a synchrotron resource for time-resolved X-ray science. J Synchrotron Radiat. 2011;18(Pt 4):658-70.

15. Otwinowski Z, Minor W. Processing of X-ray diffraction data collected in oscillation mode. Meth Enzymol. 1997;276:307-26.

16. Adams PD, Afonine PV, Bunkoczi G, Chen VB, Davis IW, Echols N, et al. PHENIX: a comprehensive Python-based system for macromolecular structure solution. Acta crystallographica Section D, Biological crystallography. 2010;66(Pt 2):213-21.

17. Emsley P, Lohkamp B, Scott WG, Cowtan K. Features and development of Coot. Acta crystallographica Section D, Biological crystallography. 2010;66(Pt 4):486-501.

18. Krissinel E, Henrick K. Inference of macromolecular assemblies from crystalline state. J Mol Biol. 2007;372(3):774-97.
