# Supplementary material for: Mitochondrial localization of Dictyostelium discoideum dUTPase mediated by its N-terminus
Source: BMC Res Notes. 2020 Jan 7;13:16. doi: 10.1186/s13104-019-4879-7 (PMC6947831; doi:10.1186/s13104-019-4879-7)
Supplement: Supplementary file 2 — Additional file 2. Metal-dependence and statistics for refinements. [file 13104_2019_4879_MOESM2_ESM.docx]

**Additional Material Table S1 and Table S2**

**Table S1**

**Metal-dependence^1^ of activities of *D. discoideum* dUTPases^2^**

| **dUTPase** | Mg^2+^ | Mn^2+^ | Ca^2+^ | EDTA |
| --- | --- | --- | --- | --- |
| full-length  (1.4 nM) | 100 | 69 ± 7 | 15 ± 4 | 4.4 ± 3 |
| core  (3.6 nM) | 100 | 77 ± 10 | 33 ± 14 | 1 ± 1 |

^1^ 2 mM final concentration of cation or EDTA

^2^  % dUMP produced relative to Mg^2+^

(average of three replicates ± standard deviation)

**Table S2.**

**Statistics for the structural refinements of the core dUTPase**^1^

| Wavelength (Å) | 0.978 |
| --- | --- |
| Resolution range (Å) | 32.63 - 2.18 (2.27 - 2.18) |
| Space group | P 21 21 21 |
| Unit cell (Å, ˚) | 71.63 72.46 75.16 90.00 90.00 90.00 |
| Total reflections | 119388 (9760) |
| Unique reflections | 20630 (1825) |
| Multiplicity | 5.8 (5.3) |
| Completeness (%) | 98.01 (87.87) |
| Mean I/sigma(I) | 20.39 (4.40) |
| Wilson B-factor (Å^2^) | 43.85 |
| R-merge | 0.09803 (0.3929) |
| R-meas | 0.1084 |
| CC_1/2_ | 0.987 (0.933) |
| Reflections used for R-free | |
| R-work | 0.2089 (0.2559) |
| R-free | 0.2510 (0.3356) |
| Number of non-hydrogen atoms | 3181 |
| macromolecules | 2920 |
| ligands | 94 |
| water | 167 |
| Protein residues | 386 |
| RMS (bonds) (Å) | 0.012 |
| RMS (angles) (˚) | 1.43 |
| Ramachandran favored (%) | 99 |
| Ramachandran allowed (%) | 1 |
| Ramachandran outliers (%) | 0 |
| Clashscore | 5.69 |
| Average B-factor (Å^2^) | 47.8 |
| Macromolecules | 47.2 |
| Ligands | 68.8 |
| Solvent | 46.6 |

^1^Statistics for the highest-resolution shell are shown in parentheses. The initial model obtained by molecular replacement using the *A. thaliana* dUTPase structure (PDB ID 4OOP) was subjected to iterative cycles of restrained refinements in PHENIX [1] and manual model building in Coot [2]. The structure was deposited in the Protein Data Bank with the ID code 5F9K.

**References**

1. Adams PD, Afonine PV, Bunkoczi G, Chen VB, Davis IW, Echols N, et al. PHENIX: a comprehensive Python-based system for macromolecular structure solution. Acta crystallographica Section D, Biological crystallography. 2010;66(Pt 2):213-21.

2. Emsley P, Lohkamp B, Scott WG, Cowtan K. Features and development of Coot. Acta crystallographica Section D, Biological crystallography. 2010;66(Pt 4):486-501.
