# Supplementary material for: Mitochondrial localization of Dictyostelium discoideum dUTPase mediated by its N-terminus
Source: BMC Res Notes. 2020 Jan 7;13:16. doi: 10.1186/s13104-019-4879-7 (PMC6947831; doi:10.1186/s13104-019-4879-7)
Supplement: Supplementary file 3 — Additional file 3: Figure S1. Temperature and pH optima. Figure S2. Secondary structure & altered positions. Figure S3. Interactions between Chains A and C. [file 13104_2019_4879_MOESM3_ESM.docx]

Additional Material Text S2 Legends Figures S1, S2, S3

**Figure S1**

**Temperature and pH optima of recombinant full-length and core dUTPases.**

Upper panel: Temperature profile; Lower panel: pH profile

Points (full-length, circles; core, squares) represent the average of three replicates of end point assays measuring dUMP produced (see Supplemental Material Text S1 Methods). Error bars indicate the standard deviation.

**Figure S2**

**Secondary structure model and altered ligand positions of the core dUTPase.**

Secondary structure model and altered ligand positions of the core dUTPase

Structure of trimeric core dUTPase (top view) with a different orientation of the ligand in each active site (insets). Subunits are colored grey (Chain A), cyan (Chain B) and light pink (Chain C), and the bound ligand (an inhibitor, dUMPNPP) is shown as a stick model. The cylinder in each subunit represents the α helix, which contains Ser98 in Motif 2 (as per UniProt ID Q54BW5; Ser65 in PDB 5F9K).

*Insets*: Close-ups of active sites viewed from the same perspective illustrate the different orientations of the inhibitor due to interactions between N-termini with the C-termini of adjacent subunits. The interaction between Inhibitor A and Ser98, shown by the black dotted line, was identified by the PISA web service [1]. The yellow circle represents Mg^2+^. Due to the lack of electron density, the location of the magnesium ion in active site A was not identified. Statistics of structural refinements are presented in SM Table 2.

**Figure S3**

**Crystal structure analyses of interactions between Chains A and C of core dUTPase.**

a. A space-filled model of the core dUTPase illustrating the orientation of Inhibitor B and the interaction of the C-terminus of Chain C (light pink) that is behind Chains A (grey) and B (cyan), with N-terminal residues of Chain A. (Circled in black; this same interaction is shown schematically in Fig. 1d.)

b. A secondary structure model of Chain C and the interacting residues from subunit A. The circle indicates the same location as shown in Panel a. For simplicity, the other subunits are not shown.

c. Rotated detailed view of the C-terminal residues of Chain C (light pink) interacting with the N-terminal residues of Chain A (grey). Residue numbers correspond to the core dUTPase polypeptide (PDB 5F9K; sequence in Fig. 1a). The black dotted lines indicate interactions obtained from the PISA interface analysis (E120::H3+F5, E123::K5 and E126::K9) [1]. Graphics were generated using The PyMOL Molecular Graphics System (Schrödinger, LLC).

**Reference**

1. Krissinel E, Henrick K. Inference of macromolecular assemblies from crystalline state. J Mol Biol. 2007;372(3):774-97.
